# Editorial: Adaptive Hot Cognition: How Emotion Drives Information Processing and Cognition Steers Affective Processing

**DOI:** 10.3389/fpsyg.2016.01920

**Published:** 2016-12-06

**Authors:** Mariska E. Kret, Bruno R. Bocanegra

**Affiliations:** ^1^Cognitive Psychology Unit, Institute of Psychology, Leiden UniversityLeiden, Netherlands; ^2^Leiden Institute for Brain and CognitionLeiden, Netherlands

**Keywords:** emotion, cognition, top-down, bottom-up, social decisions

## Introduction

Efficiently regulating our emotions can have important consequences for our survival. Whereas some situations ask for immediate action with no room left for doubts, such as when a fast-moving car is about to bump you from your bike at an intersection, other situations ask for careful consideration and reflection, such as when deciding whether to stop the life support of a beloved one. Both situations involve a life that is at risk, intense feelings and emotions and also demand a decision, albeit they cover very different timespans and differ on many other aspects as well. The current special issue includes 17 unique contributions and integrates different viewpoints on fundamental issues in the interaction between affect and cognition, gathering both empirical and theoretical contributions based on various approaches and methodologies from disciplines including cognitive psychology, social psychology, psychophysiology and neuroeconomics.

Many of the contributions we received included experimental studies where emotional manipulations were intertwined with social processes including the perception of emotions from another person or the effect of emotions on social decisions such as choosing to trust or cooperate with someone or instead pursue ones own goals and maximize personal gains. For social species like humans, effectively communicating emotions and intentions and recognizing emotion signals from others and subsequently responding with the appropriate behavior, is of pivotal importance for a healthy social life (Kret and Ploeger, 2015). Not surprisingly, most of our emotions can be associated with social events and/or decisions and this is thus clearly reflected in this special issue as well. For example, in their contribution, de Valk et al. demonstrated that being confronted with another person's facial or bodily expressions of emotion, and anger in particular, yielded faster actions in observers. This work builds on the early work by Nico Frijda, showing that all emotions are closely linked to action tendencies. He postulated that in order to attempt to better understand emotions, we should study actions rather than cognitions, feelings or emotions (i.e., Frijda et al., 1989). Although all emotions link to actions, the links for some emotions are stronger than for others. Because threatening signals are thoroughly processed (Williams et al., 2006) and rapidly detected (Hansen and Hansen, 1988; Öhman et al., 2001; Pinkham et al., 2010), they prepare for greater action preparedness (Schutter et al., 2008; Van Loon et al., 2010; Borgomaneri et al., 2014) and for quick actions (Coombes et al., 2005). As is shown in the article by de Valk et al. this is the case when processing another's facial and bodily expressions alike. Although action preparedness has traditionally been conceptualized in terms of the contrast between defensive compared to appetitive motivational tendencies, Bouman et al. demonstrate the generality of emotional influences on goal-directed action in an investigation of whole-body movement initiation in reaction to emotional pictures. They found that high arousal pictures have similar effects on forward gait initiation, independent of the valence of the picture content, which suggests that arousal is a crucial factor in explaining the link between emotion and action.

Expressions of emotion are readily picked up by observers and possibly, the mimicry of these emotional expressions might improve that even further, as Kret (2015) outlines in a review article included in this special issue. She states that, when people unconsciously mimic their interaction partner's expressions of emotion, they come to feel reflections of those companions' emotions, which in turn influence the observer's own emotional and empathic behavior. Whereas the majority of previous research focused on facial actions as expressions of emotion, this article includes research on emotion signals from sources beyond the face muscles that are more automatic and difficult to control. For example, the perception of implicit sources such as gaze and tears and autonomic responses such as pupil-dilation, eyeblinks and blushing are all very subtle reflections of a person's inner state of mind, yet they are visible to observers and because they can hardly be controlled or regulated by the sender, they have the potential to provide important “veridical” information. Interestingly, evidence is accumulating that these kinds of expressions, such as for example observing someone's pupils dilate during eye contact, without people being aware of them, do have profound effects on social decisions that people make including whether to trust someone or not (Kret et al., 2015). Interestingly, the automatic pick-up of facial and postural emotion has become a topic of increasing interest in the literature on persuasion through emotion in advertising. In their contribution, Lewinski et al. integrate the existing evidence and propose that people can resist persuasion by controlling their facial expression of emotion when exposed to an advertisement, which shows its viability as a novel and effective strategy to counteract attempts at persuasion.

Arousal, whether evoked by looking someone deeply in the eyes (Kret et al., 2015) or by hearing a frightening sound (Lee et al.) has a strong impact on emotional and cognitive processes and can have a profound positive impact on memory as well. As mentioned before, emotional information receives preferential processing, facilitating adaptive strategies for survival. But in addition, the presence of emotional stimuli and the associated arousal modify the processing of surrounding non-emotional information (Mather and Sutherland, 2011). As in the example with the biking incident described in the beginning, it is very possible that the next day at the intersection, you will be more cautious or without realizing why, even take a detour. Intriguingly, these types of decisions that are often considered “rational” can thus be driven or at least colored by unconscious affective processes. Drawing from findings from behavioral economics and neuroeconomics, Luo and Yu (2015) propose a new model of emotion and reason in decision making, which focusses on identifying factors explaining when emotions help or hurt decision making depending on the type of contexts in emotions occur. That our decisions are not always as rational as we think, even in situations that are relatively risky and where monetary gains and losses are at stake, became even more evident in an experiment by Markiewicz and Kubińska. In their study, authors provided important insights into information processing differences between affective and deliberate risk taking decisions during a card game. Specifically, they show that in a “hot” version of the Columbia Card Task, emotion interferes with correct information processing because it impairs the engagement of effortful, rule-based processes which require working memory resources. In addition, their article discusses benefits and pitfalls of different versions of the Columbia Card Task, which can benefit future studies in further disentangling affective and cognitive processing.

Markiewicz and Kubińska did not study individual differences, but if they had done so, as Steimke et al. did, they could perhaps have observed that some individuals are more prone to be put off track by emotional events than others. Especially self-control, or, the ability to exert control over ones impulses can help endure aversion, resist temptations and ignore distractions, as shown by the study by Steimke et al. Also, investigating individual differences, Zhang et al. showed that test-anxious students showed a significant deficit in executive attention when they were faced with emotional distracters, which is consistent with the idea that emotional distraction depends on an interaction between dispositional and situational factors. Over the past years, a large body of research has focused on the neural systems underlying our capacity to regulate our emotions by reappraising emotional events. Indeed, individuals may use different emotion regulation skills to modulate their spontaneous reactions. In their meta-analysis of 21 recent neuro-imaging studies Messina et al. (2015) found that modulation of neural activity not only of executive but also semantic networks may be critical for emotion regulation through reappraisal.

In this special issue, several contributions investigated emotion-cognition interactions by examining social decisions in games, such as the Ultimatum Game, Cyberball Game, Trust Game and even Tetris. For example, Ruissen and de Bruijn compared the effect of playing a cooperative vs. competitive Tetris game on a subsequent social Simon task, which measured the extent of self-other integration of action representations. Less self-other integration was observed in participants who had previously played a competitive game, compared to cooperative or solo versions of the game, which shows that the nature of the social relation between actors is sufficient to modulate social action representations. Cerit et al. (2015) used the Ultimatum Game to investigate the effect of prolonged tryptophan supplementation (a precursor of serotonin) on social cognition. Given that previous experimental manipulations indicated that serotonin increases pro-social perception, they expected to find that the supplement would lead to less rejection of unfair offers during the game. In contrast to this, however, they observed that increasing serotonin availability actually *increased* the rejection of very unfair offers, which suggests that the effects of acute vs. prolonged boosting of serotonin may have opposite effects on social cognition. Sellaro et al. (2015) also used a game setting to study the interaction between emotion and cognition. Participants in their study were exposed to social exclusion or ostracism, by observing someone else getting socially excluded. Previous research had already shown that both being ostracized and observing ostracism of another person activates the insula and the prefrontal cortex (PFC) (Dietrich et al., 2008; Kraus et al., 2013). In their experiment, Sellaro et al. investigated a putative relationship between insula-PFC activity and prosocial helping behavior toward an ostracized person during a Cyberball game. By using transcutaneous Vagus Nerve Stimulation (tVNS) they stimulated the vagus nerve and hence, indirectly boosted insula and PFC activation. In contrast to what they expected, applying tVNS did not modulate reactions to vicarious ostracism. It is always hard to interpret null-findings, yet publishing these results is of pivotal importance in order to avoid publication bias and to provide valuable insights that are helpful in designing future experiments and the authors did indeed make useful suggestions in that direction in their discussion of the results. Three of the authors involved in this study additionally wrote a second article in this issue where they discuss the effects of yet another manipulation on helping behavior (Steenbergen et al., 2015). In this study, their manipulation worked, and they showed that charitable donating could be promoted by administering the food supplement L-Tryptophan, the biochemical precursor of serotonin. Once again, it turns out that our decisions are often not rationally taken at all and this study shows that the food we eat can alter the way we think about charity and foster helping behavior. In a third study, two of the authors of the previous study performed an experiment investigating the effect of an environmental factor (an arousing “peppermint” vs. a calming “lavender” olfactory fragrance) on social interactions while performing the Trust Game (Sellaro et al., 2015). In this game, participants can transfer money to an alleged trustee in order to index interpersonal trust. Interestingly, the authors observed an increase in the money transferred due to the calming fragrance, which, consistent with the previously mentioned study, suggests that social decisions can be influenced by domain-general cognitive-control states.

In the above discussed literature, the social and emotional component were often intertwined. Whether seeing someone else's emotions, feeling another's pain whilst being excluded by others or considering another's potential monetary losses, these experiences all impact on our immediate and future behavior and decisions. That said, sometimes emotions can be triggered by very basic non-social events as well. In their study, Lu et al. investigated the affective cues present in naturalistic scenes. From an evolutionary perspective, natural scenes provide important information for observers that help them to keep safe. As is the case with social emotional cues, it is therefore also adaptive for individuals to efficiently extract such non-social information from the environment. By using a psychophysical approach and signal detection theory, authors investigated how contrast and color modulated fearfulness ratings of images of natural scenes and in order to stimulate future research, developed a fearful stimulus set including a wide range of fearfulness levels which can be controlled on a range of low-level properties. In another psychophysical study, Li and Yuen investigated a time-drag effect when participants are presented with dynamically changing sequences of facial expressions. They observed that dynamic stimulus presentations of facial expressions induced an overestimation of perceived duration, whereas overall, static presentations did not. Although this effect was observed for different expressions, it was most pronounced in angry expressions, which demonstrates that affective modulations are not only present in the spatial domain of visual perception, but extend to the temporal domain as well.

## Conclusion

The wide-range of contributions included in this special issue underscore the general conclusion that emotion plays an important role in many diverse areas of cognition including social interactions, motivational behavior, decision-making, memory, attention and perception (Dolan, 2002). From a functional point of view, the pervasiveness of emotion-cognition interactions should not be surprising considering that the underlying biological systems have presumably been continuously shaped by evolution in such a way as to promote adaptive behavior in situations that are relevant for survival or reproduction (Kret, 2015). Another key conclusion concerning the relationship between cognition and emotion that has been emerging in the field and that has been corroborated in this special issue, is that it is probably counterproductive to try to separate them (Pessoa, 2008). Instead, current thinking emphasizes their interdependence in ways that challenge a simple division of labor into separate cognitive and emotional domains and instead views the two domains as two sides of the same coin (Bocanegra, in press). We hope that bringing together this wide range of contributions increased the understanding of how emotion drives information processing and cognition steers affective processing and that the empirical evidence and theoretical ideas brought up new questions in readers and will continue to uncover exciting new avenues for future research.

## Author contributions

BB and MK conceived of the special issue and wrote the manuscript in close collaboration. We declare no conflict of interest.

## Funding

MK was supported by the Netherlands Science Foundation (VENI # 016-155-082).

### Conflict of interest statement

The authors declare that the research was conducted in the absence of any commercial or financial relationships that could be construed as a potential conflict of interest.
